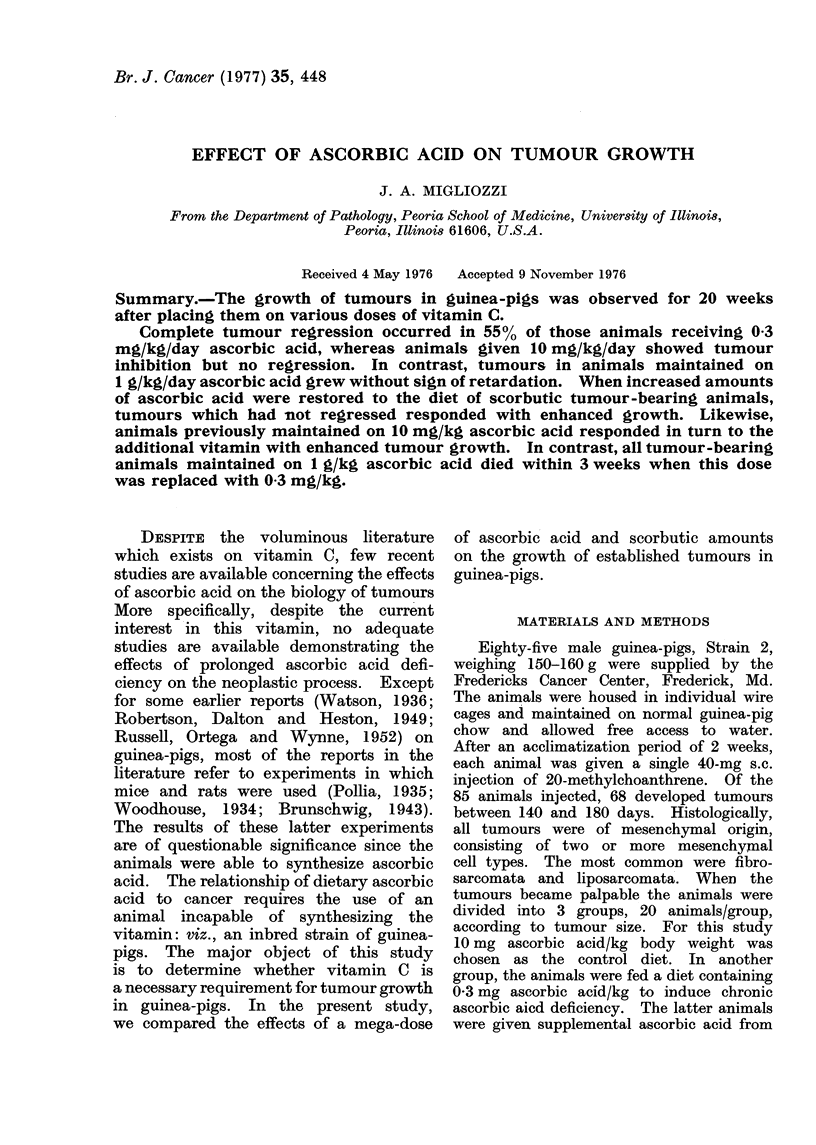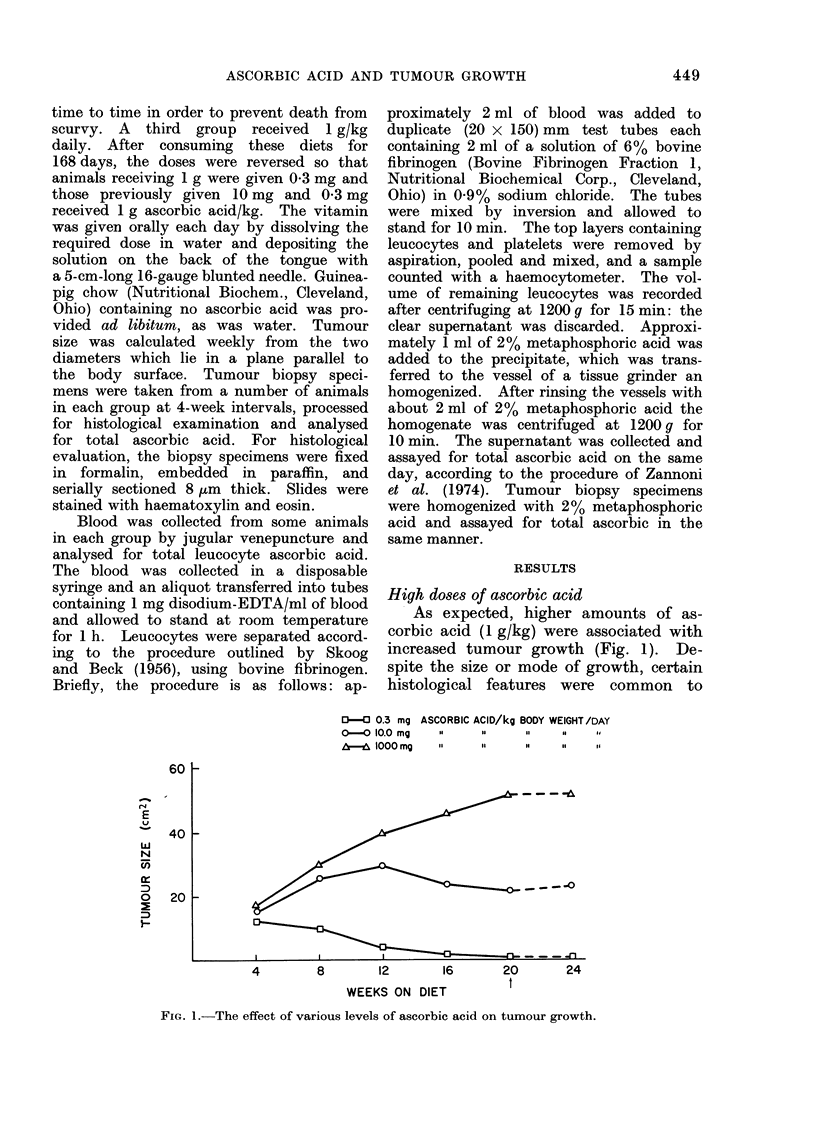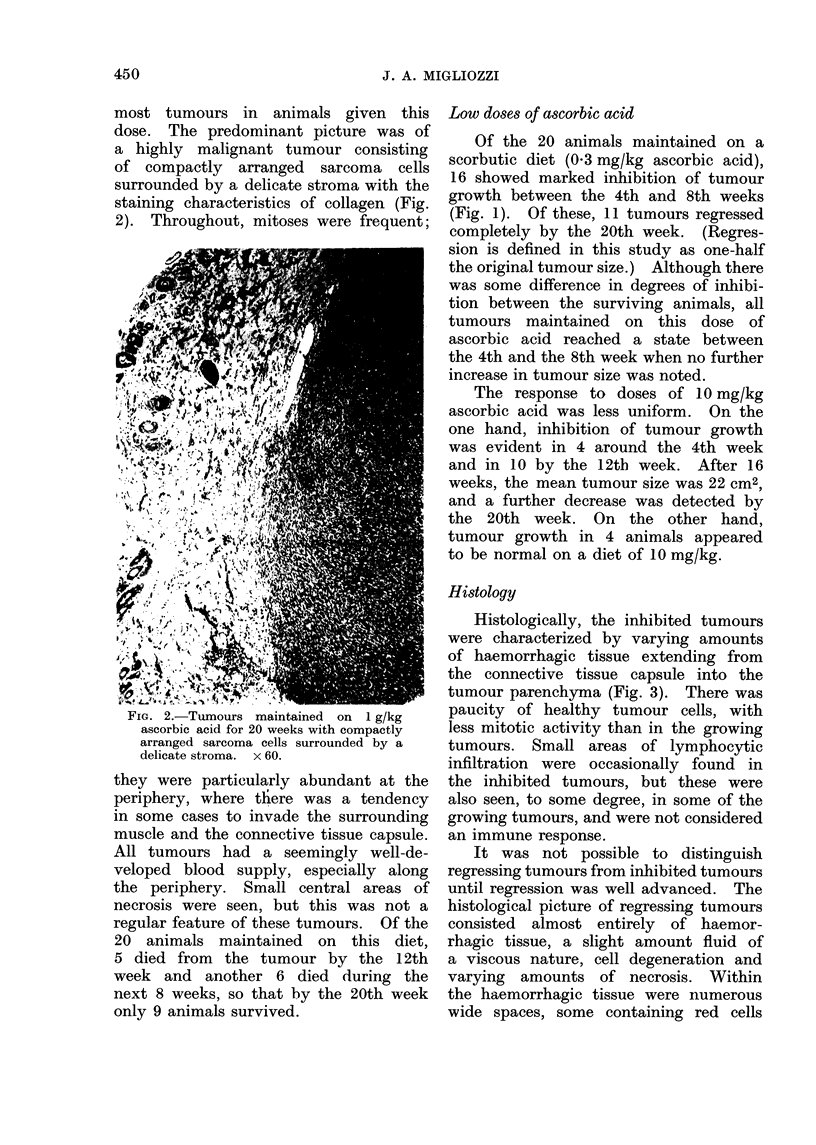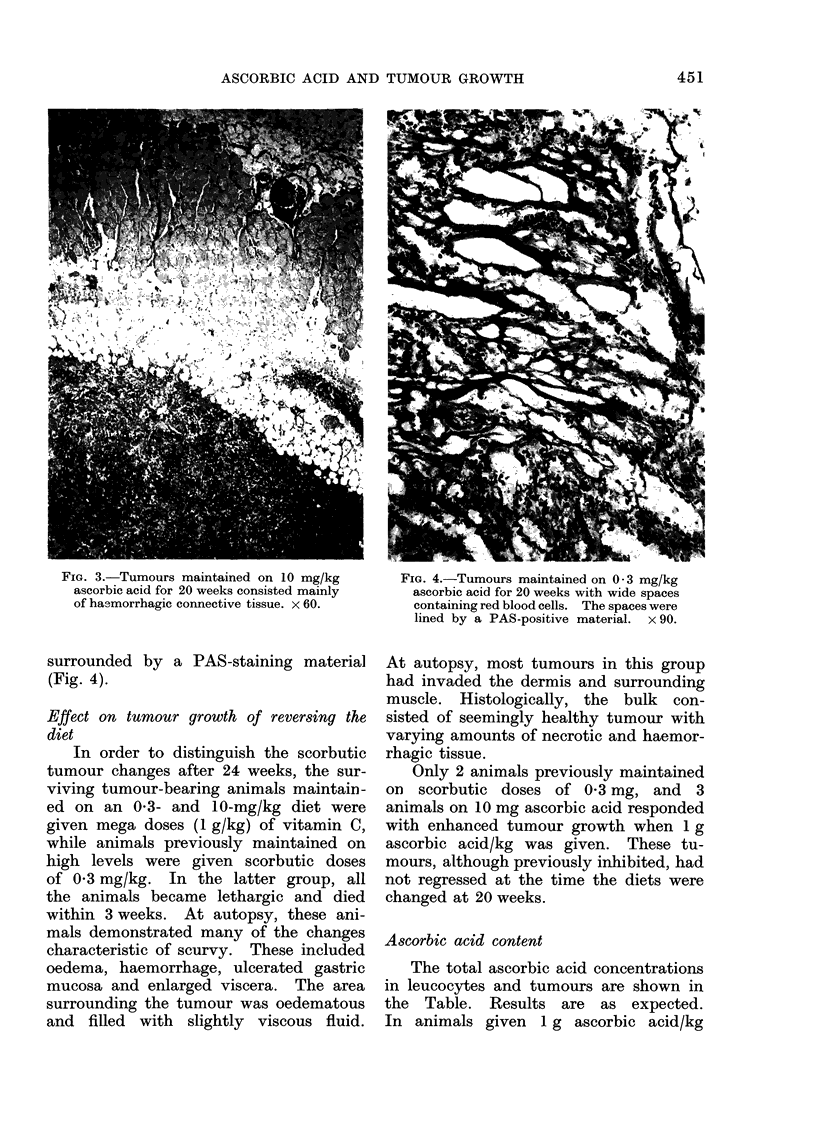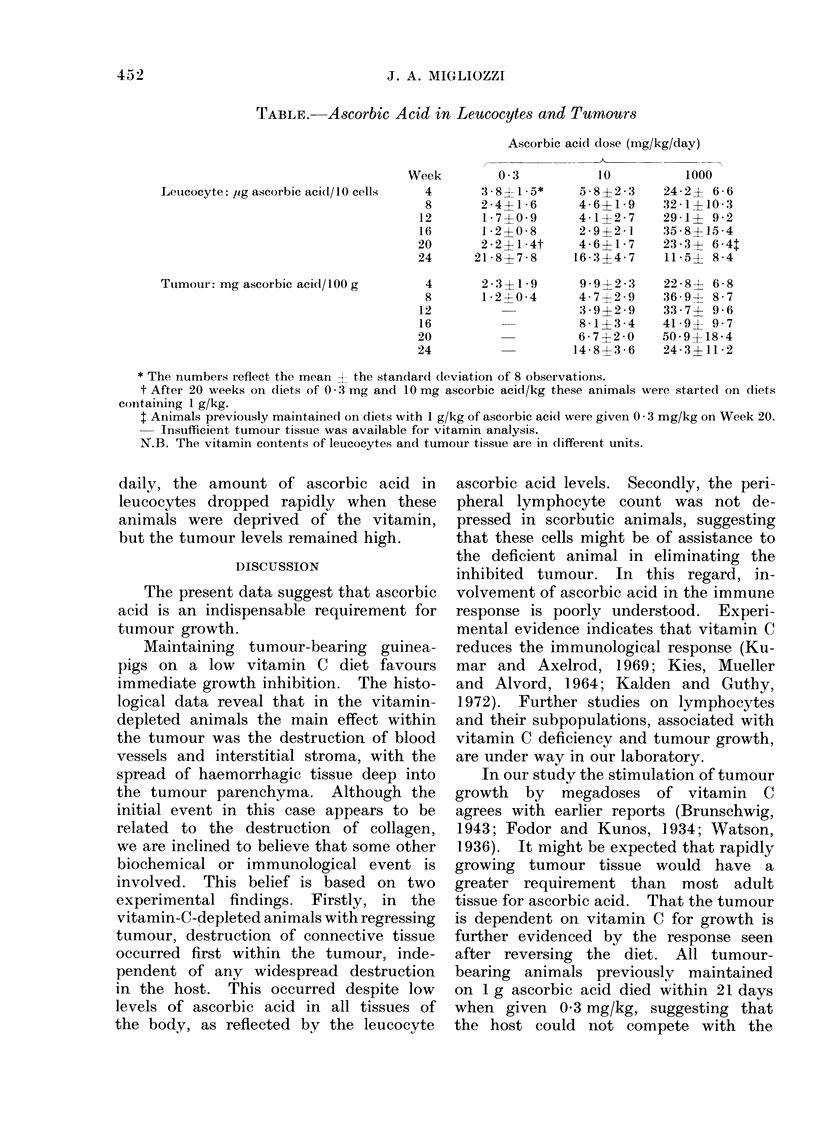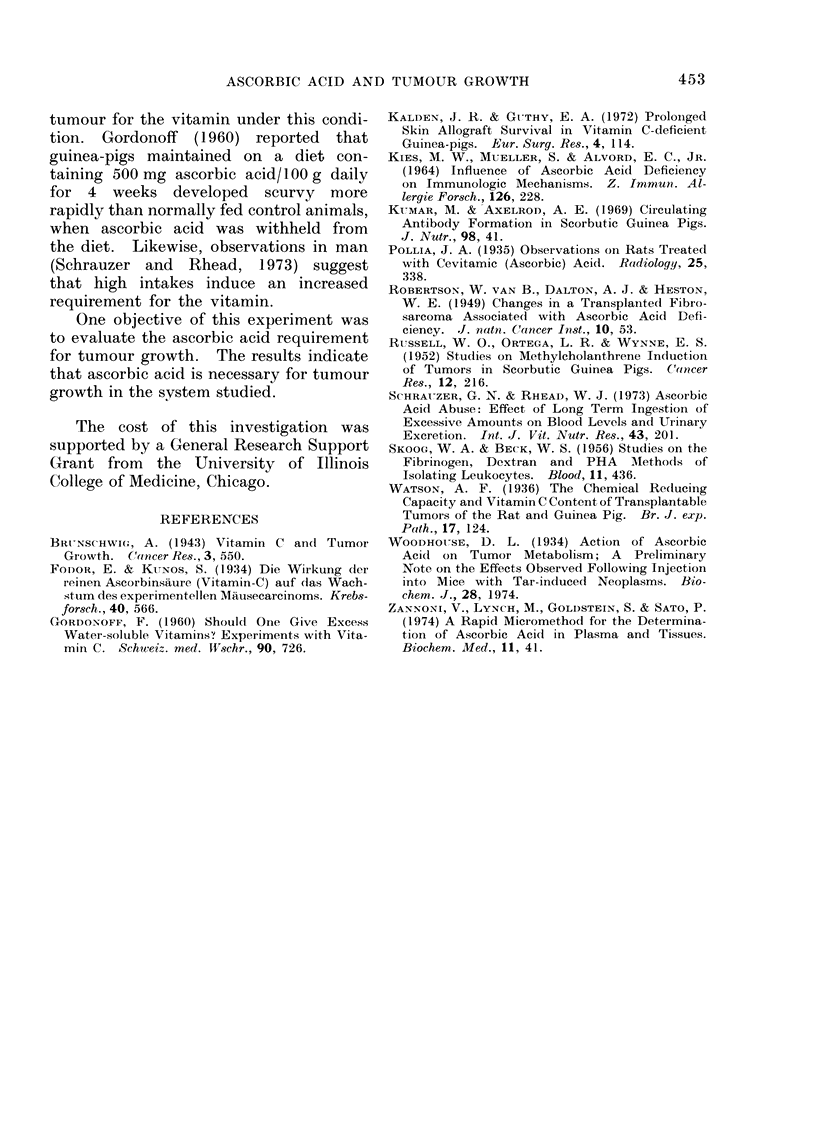# Effect of ascorbic acid on tumour growth.

**DOI:** 10.1038/bjc.1977.67

**Published:** 1977-04

**Authors:** J. A. Migliozzi

## Abstract

**Images:**


*Br. J. Cancer* (1977) **35**, 448

### EFFECT OF ASCORBIC ACID ON TUMOUR GROWTH

J. A. MIGLIOZZI

*From the Department of Pathology, Peoria School of Medicine, University of Illinois, Peoria, Illinois* 61606, *U.S.A.*

Received 4 May 1976 Accepted 9 November 1976

**Summary.—The growth of tumours in guinea-pigs was observed for 20 weeks after placing them on various doses of vitamin C.**

**Complete tumour regression occurred in 55% of those animals receiving 0·3 mg/kg/day ascorbic acid, whereas animals given 10 mg/kg/day showed tumour inhibition but no regression. In contrast, tumours in animals maintained on 1 g/kg/day ascorbic acid grew without sign of retardation. When increased amounts of ascorbic acid were restored to the diet of scorbutic tumour-bearing animals, tumours which had not regressed responded with enhanced growth. Likewise, animals previously maintained on 10 mg/kg ascorbic acid responded in turn to the additional vitamin with enhanced tumour growth. In contrast, all tumour-bearing animals maintained on 1 g/kg ascorbic acid died within 3 weeks when this dose was replaced with 0·3 mg/kg.**

DESPITE the voluminous literature which exists on vitamin C, few recent studies are available concerning the effects of ascorbic acid on the biology of tumours More specifically, despite the current interest in this vitamin, no adequate studies are available demonstrating the effects of prolonged ascorbic acid deficiency on the neoplastic process. Except for some earlier reports (Watson, 1936; Robertson, Dalton and Heston, 1949; Russell, Ortega and Wynne, 1952) on guinea-pigs, most of the reports in the literature refer to experiments in which mice and rats were used (Pollia, 1935; Woodhouse, 1934; Brunschwig, 1943). The results of these latter experiments are of questionable significance since the animals were able to synthesize ascorbic acid. The relationship of dietary ascorbic acid to cancer requires the use of an animal incapable of synthesizing the vitamin: *viz.*, an inbred strain of guinea-pigs. The major object of this study is to determine whether vitamin C is a necessary requirement for tumour growth in guinea-pigs. In the present study, we compared the effects of a mega-dose of ascorbic acid and scorbutic amounts on the growth of established tumours in guinea-pigs.

### MATERIALS AND METHODS

Eighty-five male guinea-pigs, Strain 2, weighing 150–160 g were supplied by the Fredericks Cancer Center, Frederick, Md. The animals were housed in individual wire cages and maintained on normal guinea-pig chow and allowed free access to water. After an acclimatization period of 2 weeks, each animal was given a single 40-mg s.c. injection of 20-methylchoanthrene. Of the 85 animals injected, 68 developed tumours between 140 and 180 days. Histologically, all tumours were of mesenchymal origin, consisting of two or more mesenchymal cell types. The most common were fibrosarcomata and liposarcomata. When the tumours became palpable the animals were divided into 3 groups, 20 animals/group, according to tumour size. For this study 10 mg ascorbic acid/kg body weight was chosen as the control diet. In another group, the animals were fed a diet containing 0·3 mg ascorbic acid/kg to induce chronic ascorbic aicd deficiency. The latter animals were given supplemental ascorbic acid from time to time in order to prevent death from scurvy. A third group received 1 g/kg daily. After consuming these diets for 168 days, the doses were reversed so that animals receiving 1 g were given 0·3 mg and those previously given 10 mg and 0·3 mg received 1 g ascorbic acid/kg. The vitamin was given orally each day by dissolving the required dose in water and depositing the solution on the back of the tongue with a 5-cm-long 16-gauge blunted needle. Guinea-pig chow (Nutritional Biochem., Cleveland, Ohio) containing no ascorbic acid was provided *ad libitum*, as was water. Tumour size was calculated weekly from the two diameters which lie in a plane parallel to the body surface. Tumour biopsy specimens were taken from a number of animals in each group at 4-week intervals, processed for histological examination and analysed for total ascorbic acid. For histological evaluation, the biopsy specimens were fixed in formalin, embedded in paraffin, and serially sectioned 8 $\mu$m thick. Slides were stained with haematoxylin and eosin.

Blood was collected from some animals in each group by jugular venepuncture and analysed for total leucocyte ascorbic acid. The blood was collected in a disposable syringe and an aliquot transferred into tubes containing 1 mg disodium-EDTA/ml of blood and allowed to stand at room temperature for 1 h. Leucocytes were separated according to the procedure outlined by Skoog and Beck (1956), using bovine fibrinogen. Briefly, the procedure is as follows: approximately 2 ml of blood was added to duplicate (20 × 150) mm test tubes each containing 2 ml of a solution of 6% bovine fibrinogen (Bovine Fibrinogen Fraction 1, Nutritional Biochemical Corp., Cleveland, Ohio) in 0·9% sodium chloride. The tubes were mixed by inversion and allowed to stand for 10 min. The top layers containing leucocytes and platelets were removed by aspiration, pooled and mixed, and a sample counted with a haemocytometer. The volume of remaining leucocytes was recorded after centrifuging at 1200 *g* for 15 min: the clear supernatant was discarded. Approximately 1 ml of 2% metaphosphoric acid was added to the precipitate, which was transferred to the vessel of a tissue grinder an homogenized. After rinsing the vessels with about 2 ml of 2% metaphosphoric acid the homogenate was centrifuged at 1200 *g* for 10 min. The supernatant was collected and assayed for total ascorbic acid on the same day, according to the procedure of Zannoni *et al.* (1974). Tumour biopsy specimens were homogenized with 2% metaphosphoric acid and assayed for total ascorbic in the same manner.

### RESULTS

### *High doses of ascorbic acid*

As expected, higher amounts of ascorbic acid (1 g/kg) were associated with increased tumour growth (Fig. 1). Despite the size or mode of growth, certain histological features were common to

FIG. 1.—The effect of various levels of ascorbic acid on tumour growth.

most tumours in animals given this dose. The predominant picture was of a highly malignant tumour consisting of compactly arranged sarcoma cells surrounded by a delicate stroma with the staining characteristics of collagen (Fig. 2). Throughout, mitoses were frequent;

FIG. 2.—Tumours maintained on 1 g/kg ascorbic acid for 20 weeks with compactly arranged sarcoma cells surrounded by a delicate stroma. ×60.

they were particularly abundant at the periphery, where there was a tendency in some cases to invade the surrounding muscle and the connective tissue capsule. All tumours had a seemingly well-developed blood supply, especially along the periphery. Small central areas of necrosis were seen, but this was not a regular feature of these tumours. Of the 20 animals maintained on this diet, 5 died from the tumour by the 12th week and another 6 died during the next 8 weeks, so that by the 20th week only 9 animals survived.

*Low doses of ascorbic acid*

Of the 20 animals maintained on a scorbutic diet (0·3 mg/kg ascorbic acid), 16 showed marked inhibition of tumour growth between the 4th and 8th weeks (Fig. 1). Of these, 11 tumours regressed completely by the 20th week. (Regression is defined in this study as one-half the original tumour size.) Although there was some difference in degrees of inhibition between the surviving animals, all tumours maintained on this dose of ascorbic acid reached a state between the 4th and the 8th week when no further increase in tumour size was noted.

The response to doses of 10 mg/kg ascorbic acid was less uniform. On the one hand, inhibition of tumour growth was evident in 4 around the 4th week and in 10 by the 12th week. After 16 weeks, the mean tumour size was 22 cm$^2$, and a further decrease was detected by the 20th week. On the other hand, tumour growth in 4 animals appeared to be normal on a diet of 10 mg/kg.

*Histology*

Histologically, the inhibited tumours were characterized by varying amounts of haemorrhagic tissue extending from the connective tissue capsule into the tumour parenchyma (Fig. 3). There was paucity of healthy tumour cells, with less mitotic activity than in the growing tumours. Small areas of lymphocytic infiltration were occasionally found in the inhibited tumours, but these were also seen, to some degree, in some of the growing tumours, and were not considered an immune response.

It was not possible to distinguish regressing tumours from inhibited tumours until regression was well advanced. The histological picture of regressing tumours consisted almost entirely of haemorrhagic tissue, a slight amount fluid of a viscous nature, cell degeneration and varying amounts of necrosis. Within the haemorrhagic tissue were numerous wide spaces, some containing red cells

FIG. 3.—Tumours maintained on 10 mg/kg ascorbic acid for 20 weeks consisted mainly of haemorrhagic connective tissue. ×60.

FIG. 4.—Tumours maintained on 0·3 mg/kg ascorbic acid for 20 weeks with wide spaces containing red blood cells. The spaces were lined by a PAS-positive material. ×90.

surrounded by a PAS-staining material (Fig. 4).

*Effect on tumour growth of reversing the diet*

In order to distinguish the scorbutic tumour changes after 24 weeks, the surviving tumour-bearing animals maintained on an 0·3- and 10-mg/kg diet were given mega doses (1 g/kg) of vitamin C, while animals previously maintained on high levels were given scorbutic doses of 0·3 mg/kg. In the latter group, all the animals became lethargic and died within 3 weeks. At autopsy, these animals demonstrated many of the changes characteristic of scurvy. These included oedema, haemorrhage, ulcerated gastric mucosa and enlarged viscera. The area surrounding the tumour was oedematous and filled with slightly viscous fluid. At autopsy, most tumours in this group had invaded the dermis and surrounding muscle. Histologically, the bulk consisted of seemingly healthy tumour with varying amounts of necrotic and haemorrhagic tissue.

Only 2 animals previously maintained on scorbutic doses of 0·3 mg, and 3 animals on 10 mg ascorbic acid responded with enhanced tumour growth when 1 g ascorbic acid/kg was given. These tumours, although previously inhibited, had not regressed at the time the diets were changed at 20 weeks.

*Ascorbic acid content*

The total ascorbic acid concentrations in leucocytes and tumours are shown in the Table. Results are as expected. In animals given 1 g ascorbic acid/kg

TABLE.—*Ascorbic Acid in Leucocytes and Tumours*

| | | Ascorbic acid dose (mg/kg/day) | | |
|---|---|---|---|---|
| | Week | 0·3 | 10 | 1000 |
| Leucocyte: μg ascorbic acid/10 cells | 4 | 3·8±1·5* | 5·8±2·3 | 24·2± 6·6 |
| | 8 | 2·4±1·6 | 4·6±1·9 | 32·1±10·3 |
| | 12 | 1·7±0·9 | 4·1±2·7 | 29·1± 9·2 |
| | 16 | 1·2±0·8 | 2·9±2·1 | 35·8±15·4 |
| | 20 | 2·2±1·4† | 4·6±1·7 | 23·3± 6·4‡ |
| | 24 | 21·8±7·8 | 16·3±4·7 | 11·5± 8·4 |
| Tumour: mg ascorbic acid/100 g | 4 | 2·3±1·9 | 9·9±2·3 | 22·8± 6·8 |
| | 8 | 1·2±0·4 | 4·7±2·9 | 36·9± 8·7 |
| | 12 | — | 3·9±2·9 | 33·7± 9·6 |
| | 16 | — | 8·1±3·4 | 41·9± 9·7 |
| | 20 | — | 6·7±2·0 | 50·9±18·4 |
| | 24 | — | 14·8±3·6 | 24·3±11·2 |

* The numbers reflect the mean ± the standard deviation of 8 observations.
† After 20 weeks on diets of 0·3 mg and 10 mg ascorbic acid/kg these animals were started on diets containing 1 g/kg.
‡ Animals previously maintained on diets with 1 g/kg of ascorbic acid were given 0·3 mg/kg on Week 20.
— Insufficient tumour tissue was available for vitamin analysis.
N.B. The vitamin contents of leucocytes and tumour tissue are in different units.

daily, the amount of ascorbic acid in leucocytes dropped rapidly when these animals were deprived of the vitamin, but the tumour levels remained high.

### DISCUSSION

The present data suggest that ascorbic acid is an indispensable requirement for tumour growth.

Maintaining tumour-bearing guinea-pigs on a low vitamin C diet favours immediate growth inhibition. The histological data reveal that in the vitamin-depleted animals the main effect within the tumour was the destruction of blood vessels and interstitial stroma, with the spread of haemorrhagic tissue deep into the tumour parenchyma. Although the initial event in this case appears to be related to the destruction of collagen, we are inclined to believe that some other biochemical or immunological event is involved. This belief is based on two experimental findings. Firstly, in the vitamin-C-depleted animals with regressing tumour, destruction of connective tissue occurred first within the tumour, independent of any widespread destruction in the host. This occurred despite low levels of ascorbic acid in all tissues of the body, as reflected by the leucocyte ascorbic acid levels. Secondly, the peripheral lymphocyte count was not depressed in scorbutic animals, suggesting that these cells might be of assistance to the deficient animal in eliminating the inhibited tumour. In this regard, involvement of ascorbic acid in the immune response is poorly understood. Experimental evidence indicates that vitamin C reduces the immunological response (Kumar and Axelrod, 1969; Kies, Mueller and Alvord, 1964; Kalden and Guthy, 1972). Further studies on lymphocytes and their subpopulations, associated with vitamin C deficiency and tumour growth, are under way in our laboratory.

In our study the stimulation of tumour growth by megadoses of vitamin C agrees with earlier reports (Brunschwig, 1943; Fodor and Kunos, 1934; Watson, 1936). It might be expected that rapidly growing tumour tissue would have a greater requirement than most adult tissue for ascorbic acid. That the tumour is dependent on vitamin C for growth is further evidenced by the response seen after reversing the diet. All tumour-bearing animals previously maintained on 1 g ascorbic acid died within 21 days when given 0·3 mg/kg, suggesting that the host could not compete with the tumour for the vitamin under this condition. Gordonoff (1960) reported that guinea-pigs maintained on a diet containing 500 mg ascorbic acid/100 g daily for 4 weeks developed scurvy more rapidly than normally fed control animals, when ascorbic acid was withheld from the diet. Likewise, observations in man (Schrauzer and Rhead, 1973) suggest that high intakes induce an increased requirement for the vitamin.

One objective of this experiment was to evaluate the ascorbic acid requirement for tumour growth. The results indicate that ascorbic acid is necessary for tumour growth in the system studied.

The cost of this investigation was supported by a General Research Support Grant from the University of Illinois College of Medicine, Chicago.

### REFERENCES

BRUNSCHWIG, A. (1943) Vitamin C and Tumor Growth. *Cancer Res.*, **3**, 550.

FODOR, E. & KUNOS, S. (1934) Die Wirkung der reinen Ascorbinsäure (Vitamin-C) auf das Wachstum des experimentellen Mäusecarcinoms. *Krebsforsch.*, **40**, 566.

GORDONOFF, F. (1960) Should One Give Excess Water-soluble Vitamins? Experiments with Vitamin C. *Schweiz. med. Wschr.*, **90**, 726.

KALDEN, J. R. & GUTHY, E. A. (1972) Prolonged Skin Allograft Survival in Vitamin C-deficient Guinea-pigs. *Eur. Surg. Res.*, **4**, 114.

KIES, M. W., MUELLER, S. & ALVORD, E. C., JR. (1964) Influence of Ascorbic Acid Deficiency on Immunologic Mechanisms. *Z. Immun. Allergie Forsch.*, **126**, 228.

KUMAR, M. & AXELROD, A. E. (1969) Circulating Antibody Formation in Scorbutic Guinea Pigs. *J. Nutr.*, **98**, 41.

POLLIA, J. A. (1935) Observations on Rats Treated with Cevitamic (Ascorbic) Acid. *Radiology*, **25**, 338.

ROBERTSON, W. VAN B., DALTON, A. J. & HESTON, W. E. (1949) Changes in a Transplanted Fibrosarcoma Associated with Ascorbic Acid Deficiency. *J. natn. Cancer Inst.*, **10**, 53.

RUSSELL, W. O., ORTEGA, L. R. & WYNNE, E. S. (1952) Studies on Methylcholanthrene Induction of Tumors in Scorbutic Guinea Pigs. *Cancer Res.*, **12**, 216.

SCHRAUZER, G. N. & RHEAD, W. J. (1973) Ascorbic Acid Abuse: Effect of Long Term Ingestion of Excessive Amounts on Blood Levels and Urinary Excretion. *Int. J. Vit. Nutr. Res.*, **43**, 201.

SKOOG, W. A. & BECK, W. S. (1956) Studies on the Fibrinogen, Dextran and PHA Methods of Isolating Leukocytes. *Blood*, **11**, 436.

WATSON, A. F. (1936) The Chemical Reducing Capacity and Vitamin C Content of Transplantable Tumors of the Rat and Guinea Pig. *Br. J. exp. Path.*, **17**, 124.

WOODHOUSE, D. L. (1934) Action of Ascorbic Acid on Tumor Metabolism; A Preliminary Note on the Effects Observed Following Injection into Mice with Tar-induced Neoplasms. *Biochem. J.*, **28**, 1974.

ZANNONI, V., LYNCH, M., GOLDSTEIN, S. & SATO, P. (1974) A Rapid Micromethod for the Determination of Ascorbic Acid in Plasma and Tissues. *Biochem. Med.*, **11**, 41.